# Interobserver and Intraobserver Reproducibility and Reliability of the Huashan Clinical Classification System for Hirayama Disease

**DOI:** 10.3389/fneur.2021.779438

**Published:** 2021-12-03

**Authors:** Chi Sun, Guangyu Xu, Yuxuan Zhang, Zhongyi Cui, Dayong Liu, Yong Yang, Xiandi Wang, Xiaosheng Ma, Feizhou Lu, Jianyuan Jiang, Hongli Wang

**Affiliations:** ^1^Department of Orthopaedics, Huashan Hospital, Fudan University, Shanghai, China; ^2^Department of Spine Surgery, Weifang People's Hospital, Weifang Medical University, Weifang, China; ^3^Department of Orthopaedics, Lanzhou University Second Hospital, Lanzhou University, Lanzhou, China; ^4^Department of Orthopaedics, West China Hospital, Sichuan University, Chengdu, China

**Keywords:** cervical spine, Hirayama disease, classification, reproducibility, reliability

## Abstract

**Purpose:** The Huashan clinical classification system for Hirayama disease has recently been proposed and has been found useful for diagnosis and treatment. So far, however, there has been little in-depth evaluation of its reliability. Thus, this study aimed to assess the reproducibility and reliability of the system.

**Methods:** Patients diagnosed with Hirayama disease between 2019 and 2020 were recruited. Seven spine surgeons from four different institutions, including an experienced group of three and an inexperienced group of four, were trained as observers of the Huashan clinical classification system for Hirayama disease, and these surgeons classified the recruited patients using the system. Then, 2 months later, they repeated the classification on the same patients in a different order. The interobserver and intraobserver agreement between the results was analyzed using percentage agreement and weighted kappa (κ) statistics.

**Results:** A total of 60 patients were included in the analysis. For all the observers, experienced observers, and inexperienced observers, the agreement percentages were, respectively, 78.5% (κ = 0.76), 80.0% (κ = 0.78), and 78.9% (κ = 0.77), indicating substantial interobserver reproducibility. For distinguishing typical (Types I and II) and atypical (Type III) Hirayama disease among the different groups of observers, the percentage agreement ranged from 95.6 to 98.9% (κ = 0.74–0.92), indicating substantial to nearly perfect reproducibility. For suggesting conservative treatment (Types I and III) or surgery (Type II), the percentage agreement ranged from 93.3 to 96.4% (κ = 0.81–0.90), indicating nearly perfect reproducibility. As for intraobserver agreement, the percentage agreement ranged from 68.3 to 81.7% (κ = 0.65–0.79), indicating substantial reliability.

**Conclusion:** The Huashan clinical classification system for Hirayama disease was easy to learn and apply in a clinical environment, showing excellent reproducibility and reliability. Therefore, it would be promising to apply and promote this system for the precise and individualized future treatment of Hirayama disease.

## Introduction

Hirayama disease (monomeric amyotrophy), also known as juvenile muscular atrophy of the distal upper extremity, was first reported in 12 cases by Hirayama et al. in 1959 (1). While there have been numerous reports in Asia in recent decades (2–5), a growing number of cases have recently emerged in Europe and North America (6–11). The typical clinical manifestations of Hirayama disease are muscular atrophy of the distal part in the unilateral upper extremity, without sensory deficits and pyramidal signs (1, 2, 10–14). As research into the pathogenesis and evaluation of the condition has progressed, flexion cervical magnetic resonance imaging (MRI) and neuroelectrophysiological examination have been confirmed as helpful for its early diagnosis (15). Moreover, for patients whose clinical manifestations were previously considered to be inconsistent with typical Hirayama disease, these techniques also had useful diagnostic value.

As clinically managed cases gradually increased in number, atypical patients with Hirayama disease were found to be not uncommon. A nationwide survey of Hirayama disease in Japan revealed a noticeable proportion of atypical patients among all 333 cases diagnosed. Patients with atrophy of the biceps or triceps were 25 and 49, respectively, out of the 333. A total of 64 cases showed sensory disturbance and 127 cases showed tendon reflex abnormality (2). These atypical cases were specifically included in the study and were recognized by Japanese researchers. Moreover, another study had preliminarily described the clinical and imaging features of Hirayama disease manifested particularly as muscular atrophy of the proximal upper extremities (16). Also, some patients characterized by symmetrical muscular atrophy in the distal parts of both upper limbs, which was not covered by the previous diagnostic criteria proposed by Hirayama, were reported in India (17). Recently, a 15-year-old case with wasting of the bilateral dorsal interossei muscle was described in China, indicating more severe affliction (18). These cases were not inconsistent with typical patients, to some degree, and various manifestations might need different treatments or have diverse prognoses. This clinical dilemma has driven the need for a universally accepted clinical classification system for Hirayama disease.

For all these reasons, our team decided to establish a clinician-led set of guidelines for the diagnosis and treatment of Hirayama disease and recently proposed the Huashan clinical classification system (19, 20). This new system was able to facilitate daily routine medical practice on Hirayama disease as well as oral and written communications during academic conferences. We suggested that patients with special manifestations probably needed particular treatments grounded in the guideline and classification system, and we showed that we had achieved good clinical efficacy on the patients that we treated (13, 14, 21). Although the new classification system has been gradually accepted and used by researchers and clinicians, it still needs to be further investigated and its reliability needs to be proven. Therefore, this study aimed to carry out an in-depth evaluation of the reproducibility and reliability of the Huashan clinical classification system for the Hirayama disease.

## Materials and Methods

### Huashan Clinical Classification System for Hirayama Disease

The above system was established based mainly on a retrospective analysis of 359 cases (348 male patients, 16.7 ± 2.2 years old) between 2007 and 2018. The following manifestation parameters were taken into consideration to establish the classification, especially including muscle atrophy involving the upper limbs, whether the deep reflexes of the extremities were active or hyperactive, the Hoffmann's sign and other pyramidal tract damage, whether it was accompanied by sensory dysfunction such as upper limb numbness, the location of muscle atrophy, and the progress of clinical symptoms, and electrophysiological examination within 6 months. Clinical manifestation and the severity and complexity of the disease were the key considerations.

**Figure 1:**
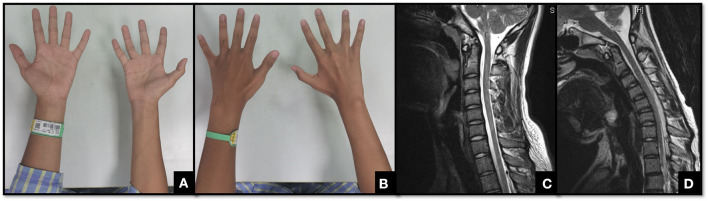
A 16-year-old male with weakness of the right hand for ~1 year. **(A,B)** show the obvious atrophy of the hypothenar and the first dorsal interosseous muscle in his right hand. **(C)** shows the spinal cord is slightly thin and without compression from C5 to C7. **(D)** shows there is noticeable cyst–wall separation behind the spinal cord in the cervical flexion position. This patient was characterized by atrophy and weakness of the unilateral upper limb, without sensory dysfunction and pyramidal signs. So, he was classified as having Type I Hirayama disease. In addition, his clinical symptoms became worse in the recent 6 months and should be determined as Type Ib.

**Figure 2:**
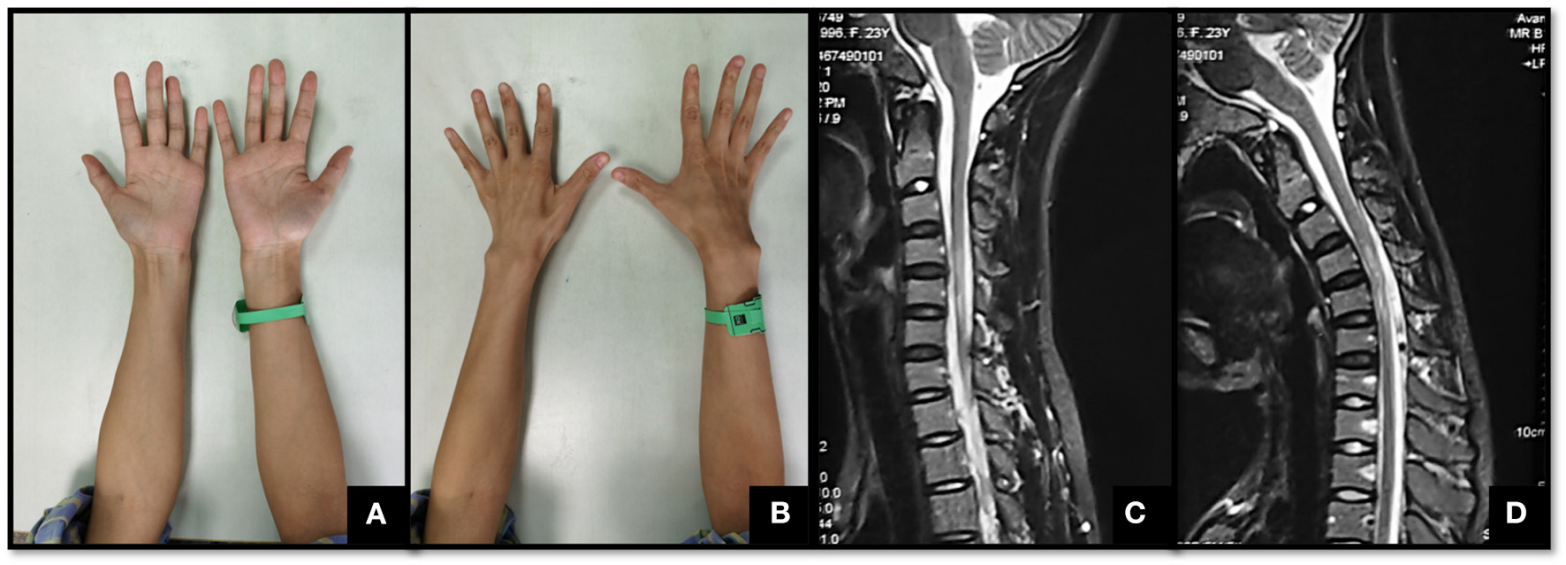
A 24-year-old female with weakness of both hands for approximately 5 years. **(A,B)** show the atrophy of the left hypothenar and the first dorsal interosseous muscles on both sides. The left upper limb was more severely affected. **(C)** shows an obviously thin spinal cord with a high signal in T2WI from C4 to C6. **(D)** shows the cyst–wall separation behind the spinal cord in the cervical flexion position. This patient was bilaterally affected, with one side more severely so. Moreover, her deep tendon reflexes were active and the Hoffmann's signs of both hands were positive. Therefore, she was classified as having Type II Hirayama disease.

**Figure 3:**
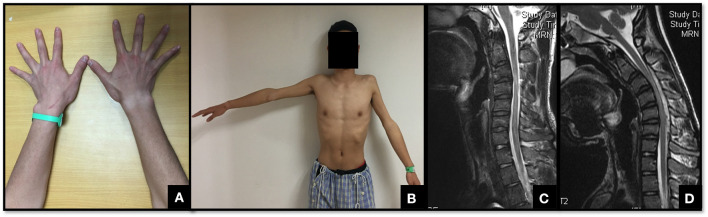
An 18-year-old male with atrophy of the bilateral upper limbs for ~1 year. There was a continued progression of weakness in the most recent 6 months. **(A,B)** show the apparent atrophy of the first dorsal interosseous muscle in the right and the deltoid in the left. The patient could not keep the left arm raised laterally for more than 10 s. **(C)** shows that the spinal cord was significantly thin from C3 to C6. **(D)** shows the cyst–wall separation behind the spinal cord in the cervical flexion position. This patient was bilaterally affected, especially in the proximal part of the left arm. His deep tendon reflexes were active and the Hoffmann's signs were positive on both sides, without sensory dysfunction. As a result, he was classified as having Type III Hirayama disease.

Consequently, the system suggested that patients with Hirayama disease should be classified into one of the following three main types: Type I, atrophy of the hand inner muscle and forearm muscle in the unilateral upper limb, or asymmetric-bilateral upper limbs (Figure 1). Then, according to whether the progress of symptoms or electrophysiological examination was seen in the previous 6 months, Type I could be divided into Type Ia (stable period) and Type Ib (progression period). Clinical treatment advice: Type Ia, regular follow-up, wearing a cervical collar if progressive, surgery if intolerant of the collar or for serious progress; Type Ib, wearing a cervical collar with regular follow-up, surgery if intolerant of the collar. Type II, atrophy of the hand inner muscle and forearm muscle in the unilateral upper limb or asymmetric-bilateral upper limbs, together with pyramidal tract damage (the deep reflexes of the extremities are either active or hyperactive, Babinski sign or Hoffmann's sign was positive) (Figure 2). Clinical treatment advice: surgery if necessary. Type III, atypical Hirayama disease, including atrophy of the proximal muscle (biceps, triceps, deltoid, supraspinatus, etc.) in the unilateral upper limb, atrophy in symmetric-bilateral upper limbs, and sensory dysfunction (Figure 3). Clinical treatment advice: wearing a cervical collar with very close follow-up, surgery if intolerant of the collar or continuously progressing (20).

### Case Selection

Patients with Hirayama disease were consecutively selected for 18 months from January 2019 to June 2020. The inclusion criteria were as follows: clear diagnosis of Hirayama disease, adolescence onset, muscular atrophy of the upper limb as the main manifestation, good cooperation with clinical examinations, receiving conservative treatment (cervical collar and follow-up), or surgery. The exclusion criteria were as follows: previous injury or surgery in the cervical spine and tumorous or infectious lesions in the cervical spine. This research study was conducted retrospectively from the data obtained for clinical purposes. We consulted extensively with the institutional review board (IRB) of Huashan Hospital affiliated with Fudan University who determined that our study did not need ethical approval. An IRB official waiver of ethical approval was granted by the IRB of Huashan Hospital affiliated with Fudan University. Informed consent was obtained from all individual participants included in the study.

### Evaluation of Reproducibility and Reliability

The detailed clinical data for the patients was summarized in Microsoft PowerPoint (PPT) (Microsoft Corporation, Washington, United States) form. In each PPT data collection, a detailed medical history (containing progression within 6 months) with a clear description of clinical symptoms and physical examination (atrophy, myodynamia, sensory, tendon reflex, and pyramidal sign) were first presented. Clear pictures of the bilateral arms and hands of the patients also were included in each data collection. X-ray, MRI, and CT images and electrophysiological reports were finally presented. Each PPT was jointly documented by two experienced doctors, who were not recruited for the subsequent observation analysis. Seven spine surgeons from four different institutions and with different clinical experience were trained on the system, and who had not previously participated in its development. These surgeons were then asked to classify the patients based on the PPT data within 6 h and to repeat the classification on the same patients in a different order 2 months later. Also, these seven spine surgeons were divided into two groups, an experienced group (three surgeons) with more than 10 years of clinical experience and an inexperienced group (four surgeons) with <10 years of clinical experience.

### Statistical Analysis

The software SPSS Statistics (version 22.0, IBM, Armonk, New York, United States) was used to test the consistency of the classification system among the different groups of observers, and the kappa (κ) statistics were calculated (22). Concerning the differences among the three main types of Hirayama disease (Ia, Ib, II, III), the linearly weighted Cohen kappa statistics were used to measure the degree of agreement, placing greater weight on the differences between observers of more than one step than on differences of only one step. The percentage agreement and kappa statistics were then calculated for: (1) the distinction between typical and atypical Hirayama disease (I, II vs. III) and (2) the distinction between conservation and surgery (I, III vs. II). The data were assessed for all 21 observer pairs and the results were averaged. The interpretation of the results was as follows: poor (κ ≤ 0.1), slight (0.1 < κ ≤ 0.2), fair (0.2 < κ ≤ 0.4), moderate (0.4 < κ ≤ 0.6), substantial (0.6 < κ ≤ 0.8), and nearly perfect (0.8 < κ ≤ 1).

## Results

### Clinical Characteristics of the Patients and Distribution of the Classification

A total of 60 patients were observed and analyzed in our study, of which 57 were males. Their average age was 18.18 ± 2.87 years, ranging from 14 to 27 years. The course of the disease was 21.05 ± 14.87 months. More than 75% of these patients were affected in the unilateral upper limb. As presented in Table 1, Type I was the most frequent type (Type Ia, 21.7%; Type Ib, 46.7%), followed by Type II (23.3%) and Type III (8.3%).

**Table 1:** The distribution of the classification.

**Observer**	**Type Ia**	**Type Ib**	**Type II**	**Type III**
1	11 (18.3%)	29 (48.3%)	15 (25.0%)	5 (8.3%)
2	9 (15.0%)	33 (55.0%)	13 (21.7%)	5 (8.3%)
3	16 (26.7%)	25 (41.7%)	15 (25.0%)	4 (6.7%)
4	15 (25.0%)	27 (45.0%)	14 (23.3%)	4 (6.7%)
5	16 (26.7%)	24 (40.0%)	14 (23.3%)	6 (10.0%)
6	13 (21.7%)	28 (46.7%)	14 (23.3%)	5 (8.3%)
7	11 (18.3%)	28 (46.7%)	15 (25.0%)	6 (10.0%)
Overall	13 (21.7%)	28 (46.7%)	14 (23.3%)	5 (8.3%)

### Interobserver Agreement

Table 2 listed the data for all observers, experienced observers, and inexperienced observers concerning the percentage agreement and weighted kappa statistics. For all the observers, the percentage agreement was 78.5% (κ = 0.76), which indicated substantial agreement. A slightly better agreement was seen for the experienced observers, and its kappa value (κ = 0.78) also indicated substantial agreement. As for the inexperienced observers, there was a similar percentage agreement of 78.9% (κ = 0.77). These close kappa values of all observers, experienced observers, and inexperienced observers clearly demonstrated the substantial reproducibility of the Huashan clinical classification system for Hirayama disease.

**Table 2:** Interobserver reproducibility.

	**All observers**		**Experienced observers**		**Inexperienced observers**	
	**Agreement**	κ^*^	**Agreement**	κ^*^	**Agreement**	κ^*^
Type Ia	85.1%	0.55	87.8%	0.65	84.7%	0.54
Type Ib	80.6%	0.61	83.3%	0.67	80.0%	0.60
Type II	95.4%	0.87	93.3%	0.81	96.4%	0.90
Type III	97.3%	0.82	95.6%	0.74	98.9%	0.92
Overall	78.5%	0.76	80.0%	0.78	78.9%	0.77
Typical vs. atypical (I, II vs. III)	97.3%	0.82	95.6%	0.74	98.9%	0.92
Conservation vs. surgery (I, III vs. II)	95.4%	0.87	93.3%	0.81	96.4%	0.90

* *weighted kappa statistics*.

As presented in Table 2, a higher level of agreement was found for the determination of typical (Type I and Type II) or atypical (Type III) Hirayama disease. The percentage agreement ranged from 95.6 to 98.9% (κ = 0.74–0.92) for all the observers, experienced observers, and inexperienced observers. This indicated substantial to nearly perfect reproducibility. As for suggesting conservative treatment (Type I and Type III) or surgery (Type II), the percentage agreement ranged from 93.3 to 96.4% (κ = 0.81–0.90), which indicated nearly perfect reproducibility. There was no obvious distinction between the experienced and inexperienced spine surgeons in terms of classification and treatment decisions.

### Intraobserver Agreement

The results of the analysis of intraobserver reliability based on percentage agreement and weighted kappa statistics were summarized in Table 3. For all seven observers, the percentage agreement ranged from 68.3 to 81.7% (κ =0.65–0.79), indicating substantial reliability. The level of agreement was almost the same as that for the interobserver agreement.

**Table 3:** Intraobserver reliability.

**Observer**	**Agreement**	κ^*^
1	80.0%	0.75
2	81.7%	0.79
3	78.3%	0.75
4	75.0%	0.74
5	68.3%	0.65
6	71.7%	0.70
7	75.0%	0.75
Mean	75.7%	0.73

* *weighted kappa statistics*.

## Discussion

Owing to the development of neuroelectrophysiological examination and flexion cervical MRI, increasing numbers of patients with Hirayama disease have been identified. The more cases we managed, the more diverse clinical manifestations we encountered. In addition, there is no definite consensus on the treatment of Hirayama disease. In the past, a cervical collar was widely used to restrict the flexion of the cervical spine of patients, which achieved a reasonable therapeutic effect (23, 24). Currently, physicians believe that a cervical collar can alleviate the disease effectively until it reaches the stage of self-limitation, especially for patients with a relatively short disease course and slight atrophy of the spinal cord (25, 26). However, some patients wore the collar for an excessive length of time if they were diagnosed in the early stages of the disease. Parts of the patients were disabled in order to continuously bear this treatment because of unsatisfactory compliance. As a consequence, researchers attempted to perform surgery on patients using different operating methods, such as posterior cervical duroplasty, anterior cervical decompression and fusion, anterior cervical internal fixation, and posterior cervical long-segmental internal fixation. Nevertheless, the indications of those operations showed apparent discrepancies in the literature (13, 14, 27–30).

Due to the clinical dilemmas described above, the choice of scientific preoperative evaluation and a reasonable treatment strategy was particularly important. A classification system for Hirayama disease was urgently needed, which would not only guide clinical practice but also facilitate both oral and written communications at academic conferences and in clinical studies. A comprehensive and effective classification system should have the following characteristics: (1) generality, including all types of Hirayama disease; (2) reasonability, simple and easy to remember and practice; (3) guiding ability, proposing treatment options for each type; and 4) consistency, including good reproducibility and reliability. As such a classification system scarcely existed, we recently established the Huashan clinical classification system for Hirayama disease on the basis of the clinical manifestations of patients (20). In this system, we particularly suggested that surgery for Hirayama disease must be strictly fit for some indications, and not all patients should receive surgery. The three main types of the disease showed a somewhat progressive relationship based on clinical severity, which could help to guide appropriate treatments.

According to the results of this study, the new classification system showed interobserver and intraobserver agreement at a sufficient level to serve as a reliable method for clinical practice on Hirayama disease. The interobserver variability was assessed between observers with different levels of experience who worked at different institutions. Considering the fact that seven spine surgeons from four different institutions and with different clinical experiences were only trained on the system but had not developed it, the percentage agreement of 78.5% with a kappa value of 0.76 indicated a high level of agreement. This meant that the classification system was simple enough to understand and apply consistently. The average levels of agreement among all the observers, experienced observers, and inexperienced observers showed only very small differences, also suggesting that the system was easy to learn. As our study included consecutive patients from our hospital, the interobserver agreement might be further enhanced by providing a larger number of representative and educational cases of each type. In addition, we creatively studied the agreements between typical and atypical presentations and between those suggesting conservative treatment and those needing surgery. This was because identifying atypical Hirayama disease and giving scientific treatment strategies were crucial in a clinical setting. Our results gave satisfactory agreements in this regard as well. As for intraobserver variability, the percentage agreement of 68.3 to 81.7% (κ = 0.65–0.79) indicated that the classification system was stable enough to apply consistently. The substantial kappa values of both interobserver and intraobserver agreement suggested that the system was highly reproducible and reliable.

In the Huashan clinical classification system for Hirayama disease, common and unusual clinical manifestations were both taken into account. It is particularly necessary to point out that we suggested that patients with different classification characteristics should be classified into a higher priority type. For example, if one patient had positive pyramidal signs and sensory dysfunction simultaneously, then they should be classified as Type III priority. In our study, 19 of 69 patients presented a positive Hoffmann's sign and active or hyperactive tendon reflexes, but only 14 of 19 were finally regarded as Type II. For this type of patient, operations should be more proactively performed to avoid further impairment of the spinal cord. On the other hand, we needed to be more cautious about Type III Hirayama disease. Close follow-up was necessary, and the time to surgery was based on the stage of disease, compliance, and whether motor neuron disease could be ruled out. We had emphasized similar viewpoints in our previous guidelines on Hirayama disease (19).

To the best of our knowledge, no other researchers have previously focused closely on the classification of Hirayama disease, but there have already been some reports of atypical cases, which could be classified into one of the types we have proposed. Based on the data from the Japanese nationwide survey in 2006, we speculated that at least 64 of the 333 cases could be classified as Type III in terms of sensory disturbance. At least 127 of the 333 cases should have been preliminarily classified as Type II because of the common tendon reflex abnormality in the survey (2). In another report regarding 106 patients with Hirayama disease investigated in India, approximately 10% of those patients presented bilateral symmetric involvement, which could be regarded as Type III based on our classification system. The bilaterally symmetric Hirayama disease was raised as a severe form of a classic disease that might remain undiagnosed in some cases due to a common notion that it was a unilateral or grossly asymmetric disease (17). Liu et al. reported a 15-year-old boy who presented with a progressive left to symmetric-bilateral hand weakness and cold paresis over 1 year (18), which should be classified as Type III Hirayama disease. However, no reasonable treatment plan was given in their case report. We suggested a very close follow-up and the wearing of a cervical collar, with the option to receive surgery if necessary. Based on an analysis of previous atypical cases, we believe that the new classification system is helpful for covering all kinds of clinical cases and is expected to guide subsequent treatment.

There were several limitations to our study. First, the classification system was proposed, applied, and evaluated by the same institution, mainly because of its being one of the largest clinical centers for Hirayama disease in the world, where there might have been bias as to how the statistics were obtained and/or analyzed. Therefore, we invited seven observers with various levels of clinical experience from four different institutions to conduct the assessment. Moreover, these observers had not previously participated in the establishment of the system, which we believed improved the quality of the statistical analysis. Second, the classification system proposed in this study was based mainly on clinical manifestations and progression and did not include specific imaging and electrophysiological indices, which need to be further improved in subsequent studies. Third, treatment recommendations based on the classification system were not verified by long-term regular follow-up of cases, so it would be prudent to apply and promote this system in clinical settings in the future.

## Conclusions

The Huashan clinical classification system for Hirayama disease filled the blank in the field of classification of this disease. The system has excellent reliability and provides valuable guidance for clinical intervention.

## Data Availability Statement

The raw data supporting the conclusions of this article will be made available by the authors, without undue reservation.

## Ethics Statement

The studies involving human participants were reviewed and approved by The Institutional Review Board (IRB) of Huashan Hospital affiliated to Fudan University. Written informed consent to participate in this study was provided by the participants' legal guardian/next of kin. Written informed consent was obtained from the individual(s) for the publication of any potentially identifiable images or data included in this article.

## Author Contributions

CS: acquisition of data, interpretation of data, and drafting of the manuscript. GX: acquisition of data and analysis of data. YZ, ZC, DL, YY, and XW: acquisition of data. XM: material support. FL: administrative support. JJ and HW: conception and design and were co-senior authors. CS and GX were co-first authors. All authors contributed to the article and approved the submitted version.

## Funding

This study was funded by the Clinical Research Plan of Shanghai Hospital Development Center (HW, No. SHDC2020CR4030), Clinical Technology Innovation Project of Shanghai Hospital Development Center (HW, No. SHDC12019X26), National Natural Science Foundation of China (JJ, No. 82072488), and AO Spine National Research Grant 2020 (HW, No. AOSCN(R)2020-09).

## Conflict of Interest

The authors declare that the research was conducted in the absence of any commercial or financial relationships that could be construed as a potential conflict of interest.

## Publisher's Note

All claims expressed in this article are solely those of the authors and do not necessarily represent those of their affiliated organizations, or those of the publisher, the editors and the reviewers. Any product that may be evaluated in this article, or claim that may be made by its manufacturer, is not guaranteed or endorsed by the publisher.
